# Racial Disparities and COVID-19: the Social Context

**DOI:** 10.1007/s40615-021-00988-8

**Published:** 2021-03-17

**Authors:** Cora B. Marrett

**Affiliations:** Department of Sociology, University of Wisconsin-Madison, Madison, WI USA

**Keywords:** Data inadequacies, Racism and health, Trust and vaccines, Potential of collaborations

## Abstract

The coronavirus continues to take a devastating toll on the population of the USA. But that toll is not identical across all segments of the population. Specifically, Black citizens are more likely than their White counterparts to experience the dislocations associated with the coronavirus. Nor is the extent of racial differences fully known, given limitations to the testing, hospitalization, and other data currently compiled. What does emerge, however, is an understanding that the reported outcomes reflect social inequities rather than biological predispositions. The inequities flow from both historical forces and contemporary ones that leave sizeable fractions of the Black population without access to quality healthcare or safe environments. The discrepancies suggest that the development of safe and effective vaccines might not eliminate the racial disparities associated with COVID-19, for that development will not alone erode levels of structural racism in the society. Concerted actions that engage multiple segments and participants are demanded.

## Overview

The USA recorded in September of 2020 that over 200,000 persons had died from COVID-19. One source after another lamented this development, noting that it placed the nation outside of the range for other countries and in fact represented an undesirable milestone. If the numbers revealed a catastrophe for the country as a whole, for subpopulations, the numbers were even more devastating. Available sources unveil sharp differences across racial and ethnic lines in experiences with the novel coronavirus. Black Americans are far more likely than their fellow White citizens to appear in statistics on COVID-19 infection as well as death rates. As disconcerting as those statistics might be, they do not necessarily illustrate the extent to which the pandemic has ravaged the nation or generated appreciable disparities within it.

This discussion summarizes available information on race and COVID-19, paying particular attention to gaps in recorded figures, and explanations for reported disparities across racial groups. The reasons highlighted are those centered on the social context which Black and White citizen encounter. In considering efforts made or needed to reduce the inequities, the discussion highlights in particular the roles that the National Academies of Sciences, Engineering and Medicine (NASEM) might play. The focus on NASEM reflects the urgent need for knowledge-based efforts to improve data, illuminate racism, and promote public policies that advance the well-being of all. NASEM has signaled its interest in bringing the expertise of the science, engineering, and medical community to move the nation closer to this goal.

## On COVID-19 and Racial Disparities

Sources converge on this conclusion: Racial and ethnic minorities in the USA bear disproportionately the burdens of COVID-19. Those sources show higher infection, death, and hospitalization rates, even when the rates take account of differences in the age profiles of populations. The rates for Indigenous peoples, non-white Hispanic or Latinx residents, and Black persons outstrip those for non-Hispanic Whites. The present discussion focuses on Black Americans, to avoid overgeneralizing about all ethnic groups and to recognize historical and related conditions rather specific to the Black experience. Additionally, the nationally available data on groups classified often as “other” are extremely limited or cover disparate groups. The category sometimes—but not always—incorporates Indigenous peoples, Asian Americans, and Native Hawaiians or Pacific Islanders.

The death rates from COVID-19 for Black Americans far exceed those for Whites. Working with data from across the USA, the APM Research Lab has found a mortality rate among Blacks that doubles the rate for Whites. Aware that COVID-19 deaths vary, depending on age, the researchers examined the results by race and age. Importantly, racial contrasts remained, even for comparable age groupings. In fact, in every age grouping, deaths among Blacks exceeded those for Whites.^1^

These statistics, although sobering, undoubtedly understate the ravages of COVID-19 on Black Americans. Inaccuracies and incompleteness plague the national data on deaths from COVID-19. Data from specific locales reinforce the picture on disparities, however. Dane County—site of the capital of Wisconsin—has reported that Blacks account for 18.6% of the known cases and 12.5% of the deaths. Yet, Blacks comprise only 5.5% of the population in the city. At the state level, the COVID-19 death rate for Blacks is nearly 5 times the overall rate. Maryland reported recently that Blacks accounted for 37,000—or nearly 32%—of the COVID cases in the state. They constituted just under 30% of the statewide population. Even more stark: Blacks comprised 40% of the deaths in the state attributed to COVID-19.^2^

The story of confirmed infections mirrors that on COVID-19 deaths. Basically, measures of confirmed cases reveal significant contrasts between Blacks and Whites. As with the data on deaths, the state level data on confirmed cases are incomplete.^3^ Even the incomplete data undoubtedly understate disparities, for they come from people who have been tested. Yet, not all infected persons display symptoms; and those who do not are unlikely to be tested.^4^ Variations by race in screening and testing affect how validly infections data capture racial disparities.

Understanding the extent of these disparities requires refinement in the information compiled on infection rates. The same conclusion applies to another widely used measure: Rates of hospitalization. Hospitalization rates overlook the array of non-medical conditions potentially affecting the rates. Among those determinants: the availability of insurance and what treatment a physician might recommend.

Understanding more completely how COVID-19 impacts racial communities differentially requires improvements in the statistics that are gathered and reported. This can demand disaggregating data, to consider how the numbers vary for racial groups—and even among segments within them. Not surprising, then, is the frequency with which recommendations for improving data on race and COVID-19 emerge. But the recommendations center on more than COVID-19 statistics. They highlight the circumstances that appear to account for the discrepancies, evident even the incomplete data. The sources lie in the social worlds which Black Americans traverse.

## The Social Context of Racial Disparities

The data on racial discrepancies clearly are limited in many respects. Yet, experiences with the pandemic confirm what research on health long has shown. The COVID-19 disparities ensue from a range of social inequalities that have produced racial differences in life chances. Those inequalities, not genetic or biological traits, emerge as key explanations for differences in infection and death rates. Although COVID-19 is often associated with health problems, such as type 2 diabetes, those problems generally flow from unhealthy living or working conditions, along with encounters in the broader society. We know from an abundance of epidemiological studies that crowded residential and work settings affect the spread and severity of disease. We know, too, that sizeable numbers of Black Americans live in multigenerational settings and work in close quarters with other persons. Access to safe places, healthy food, and medical care matters—but eludes large numbers of Blacks. Such access requires command of economic resources. The evidence is clear, however: Blacks and Whites differ noticeably in the economic resources they control—and particularly those related to wealth.

An array of sources links social conditions to pandemic-related outcomes. But COVID-19 can also alter substantially what Blacks experience. Nor are the experiences found only among persons who are infected. The consequences can be felt throughout groups and communities. When education systems rely on technology, to reduce person-to-person contacts, all students are affected. The constraints on learning might be particularly evident for those who lack the infrastructures—technological and support systems—needed for remote learning. Again, the orbit encircles far more than the pupils in households with COVID-19 cases.

Another line inquiry proposes that the coronavirus could sharpen existing inequalities—through its effects on economic activity and thus on employment. More than one analysis speculates that increases in unemployment could result as well in heightened rates of poverty. Such an outcome could be devastating for the Black population, in light of the burdens poverty already imposes. Now, the effects of COVID-19 on economic well-being could be altered through the creation of public programs enacted to blunt economic dislocations. This suggests that responses to COVID-19-related disparities should not center exclusively on the realm of health, for healthiness might well depend on what is available in the larger social context.

## On Systematic Racism and Health Disparities

The disparities, generated or laid bare by the pandemic, have been attributed to race-based biases or racism. Racism assumes that the physical characteristics found in a population signify underlying mental and related capacities. This assumption creates opportunities for some persons and constrains them for others. Racism, then, assigns value in ways that promote unequal chances.^5^ The disparities associated with COVID-19 do not derive from random forces but result instead from the adverse behaviors and circumstances that Black Americans confront.

The founder of the Black Doctors COVID-19 Consortium makes the case this way: “The virus does not discriminate; however, the people in the institutions that control whether and if you are treated for coronavirus may discriminate – and it has the same deleterious effects on the African-American community.”^6^ Supporting the view that discrimination plays a part is the finding that doctors are less likely to refer Blacks, than Whites, for testing even when the patients in fact exhibit comparable symptoms.

Early in 2020, the city of Milwaukee took an action other communities would embrace: It declared racism as a public health crisis. The declaration emerged, based on the tracking of COVID-19 cases. The tracking showed far higher rates of confirmed cases among Blacks than among Whites. Milwaukee is not alone in linking COVID and health disparities to racism. Several states and local jurisdictions have reviewed their own trends, uncovered racial differences, and identified racism as the underlying force.

Racial inequities in the healthcare system are not new. At times, the inequities have appeared in the use of Black subjects for experimentation. Although the Tuskegee syphilis study is one of the more widely cited works on race and experimentation, it by no means stands alone in the historical record (see Washington 3, and Gamble 1.) The record includes as well residential segregation plans, set forth to protect White Americans from the health “scourges” found among Black residents. In the early twentieth century, political figures in Baltimore, cognizant of sharp racial differences in tuberculosis, cholera, and other infectious diseases, proposed schemes that would remove Blacks from areas with White citizens. Those plans, and similar developments, help explain the spatial patterns now found for race and COVID-19.^7^ The patterns, often reflected in measures of residential segregation, frequently result in restricted access to healthcare facilities and heightened exposure to infectious diseases.

Not every declaration regards discrimination within the healthcare system as the source of disparities. Rather, racism can have its consequences through the stress people encounter as they negotiate their day-to-day lives. An array of epidemiological studies highlights the unhealthy results associated with inadequate access to safe places, healthy food, and clean air. Navigation in dangerous, polluted places—that often are food deserts—can unquestionably impose strains with far-reaching consequences. Given that racism can have widespread and long-lasting consequences, it demands systematic analyses and not simply acknowledgement that it probably matters.

## Towards an Agenda for Action

Clearly, unveiling and reducing the disparities associated with COVID-19 require a multi-pronged approach. There are obvious needs for reliable and valid data. In addition, the context in which COVID-19 is occurring merits detailed study. That context is not experienced identically, even across a given population group. The character and weight of racism deserve more than cursory treatment. Decision-makers—in government, business, and academe—need evidence-based strategies that promise to protect the well-being of all.

A consortium of organizations has called on Congress and the Administration to initiate a high-level, comprehensive review of needs associated with COVID-19. The consortium recommends that the Congress assigns the responsibility to a qualified, non-partisan entity “such as the National Academies of Science, Engineering, and Medicine.” The proposal envisions a broad-gauged approach, including “medical research and development, social, behavioral and economic considerations, corporate partners to ensure product and service delivery, small businesses, universities and research institutions, as well as healthcare professions.”^8^

The aggregation of expertise to address problems and reduce disparities does appear to be an appropriate role for NASEM. The three Academies have aggregated information on the COVID-19-centered activities underway throughout the complex. This includes reports from the Societal Experts Action Network (SEAN) on limits to the data about the coronavirus as well as research findings emerging from the social and behavioral sciences. The National Academy of Engineering has summoned its members to make COVID-19 an urgent concern. The Board on Science Education, in concert with other NASEM entities, has reviewed school-related challenges surrounding the pandemic and outlined possible responses by administrators and others. A Committee on Emerging Infectious Diseases and 21st Century Health Threats has issued a series of reports addressed to questions policy-makers face.

The discussions illustrate widespread interest within NASEM in enhancing data, sharpening conceptual matters, and advancing policies to reduce the dislocations the pandemic has wrought. Importantly, the efforts frequently center on the dislocations and disparities associated with race. The Roundtable on the Promotion of Health Equity, for example, has surveyed demographic statistics on COVID-19 and uncovered glaring fissures in what is reported about racial disparities. The Roundtable on Black Men and Black Women in Science Engineering and Medicine has contributed analyses on racism and the coronavirus along with roles that public health agencies, medical schools, and athletic entities, among others, might play. The Government-University-Industry Research Roundtable has recognized the disproportionate impact of the pandemic on Black and Latinx communities and outlined plans to understand it. To the Board of Population Health and Public Health Practice, certain patterns in the society in turn affect experiences with the pandemic. To this Board, racial residential segregation must be targeted, if the health of all American is to be improved.

The interest in the well-being of the population, coupled with the commitment of NASEM leadership to diversity and inclusion, should indeed enable the building of a robust, resilient nation for all citizens. The leadership has pledged to “strive for a culture of inclusion” in all of the organization’s activities.^9^ At the request of Congress, NASEM has launched a major study on the effects of racism across science, engineering, and medicine. If that pledge for inclusion and a broad examination of racism promotes cooperation throughout the science, engineering, and medical communities, then the result could be the building of resilient systems that embrace all.

## Footnotes

1 “The Color of Coronavirus: COVID-19 Deaths by Race and Ethnicity n the U.S.” https://www.apmresearchlab.org.covid/deaths-by-race (September 16, 2020)

2 Madeleine O’Neil, “Outreach to Minority Communities Continues as COVID-19 Disparities Persist.” https://www.heraldmailmedia.com/news/special/coronavirus/outreach-to-minority-communities-continues-as-covid-19-disparities-persist

3 See information from Johns Hopkins University. https://coronavirus.jhu.edu/data/racial-data-transparency (September 29, 2020)

4 The Center for Disease Control and Prevention estimates that 40 percent of infected persons are asymptomatic.

5 See especially Camara Jones, “Naming Racism,” https://www.radcliffe.harvard.edu/event/2020-naming-racism.

6 See Ala Stanford’s response to “The Color of Coronavirus,” https://www.apmresearchlab.org/covid/response-panel#stanford. A similar argument appears in: Ella St George Carey, “Most Medical Professionals Aren’t Racist but our Medical System is,” https://www.washingtonpost.com/outlook/2020/05/18/most-medical-professionals-arent-racist-but-our-medical-system-is/.

7 Lawrence Brown (2020) contends that discussions on health disparities underplay the significance of racial segregation. Because of such segregation, high rates of COVID-19 are not randomly distributed geographically. See his summary in https://www.nationalacademies.org/event/04-28-2020/webinar-pursuing-data-on-covid-19-the-health-inequity-multiplier.

8 Letter to Chairs and Ranking Members of House Appropriations Committees, March 30,2020

9 The three presidents have issued a statement on diversity and inclusion, assuring their determination to highlight these values in both the internal and external activities of the organization. See https://www.nationalacademies.org/about/institutional-policies-and-procedures/statement-on-diversity-and-inclusion.
